# Monodisperse spherical Ag^+^ doped Cs_2_KBiCl_6_ nanocrystals: utilizing steric hindrance engineering for inkjet printing of anti-counterfeiting patterns†

**DOI:** 10.1039/d4na00988f

**Published:** 2025-05-06

**Authors:** Shaoli Song, Youlun Zhu, Hanmei Jiang, Huichao He, Qian Yang, Jianbei Qiu, Tao Han

**Affiliations:** a College of Materials and New Energy, Chongqing University of Science and Technology Chongqing 401331 China danbaiht@126.com; b School of Materials Science and Engineering, Kunming University of Science and Technology Kunming Yunnan 650093 P. R. China

## Abstract

Anti-counterfeiting is one of the critical application fields of luminescent materials. Nanocrystal luminescent materials are more suitable for anti-counterfeiting applications because they are tiny and more conducive to patterning. The preparation of monodisperse and spherical lead-free perovskite nanocrystals by a propagable method is a hot topic in anti-counterfeiting materials. In this work, monodisperse spherical Ag^+^ doped Cs_2_KBiCl_6_ nanocrystals with an average diameter of 3.51 nm were prepared by using the propagable ligand-assisted reprecipitation method at room temperature, attributed to steric hindrance engineering by increasing the ligand size. Due to relaxation of the transition barrier due to doping, excited with 365 nm ultraviolet light, the nanocrystals exhibit orange emission peaking at 600 nm, which is related to the transition recombination of Bi^3+^ (^1^S_0_ → ^3^P_1_), and the maximum photoluminescence quantum yield is 3.80%. A printable ink is prepared by combining the nanocrystals with PDMS adhesive and its curing agent, which can be used for inkjet printing of anti-counterfeiting patterns.

## Abstract 2

Monodisperse spherical Ag^+^ doped Cs_2_KBiCl_6_ nanocrystals are used to prepare an inkjet printable ink for anti-counterfeiting patterns.

## Graphical abstract

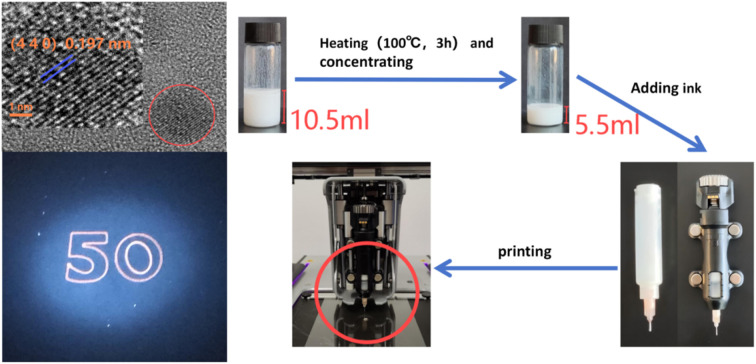

## 1 Introduction

The patterning of luminescent materials is a vital part of its application in anti-counterfeiting.^1–5^ Compared with micron-sized crystal luminescent materials, nanocrystalline luminescent materials are more suitable for patterning due to their tiny size.^6–10^ Combining nanocrystal luminescent materials with organic solvents to prepare printable inks has been proven to be a reliable solution for their patterning.^11^ At present, the popular nanocrystal luminescent materials for anti-counterfeiting are lead halide perovskite nanocrystals prepared by the hot injection method. However, the hot injection method has the following disadvantages: the obtained nanocrystals are usually square and poorly dispersed, which is not conducive to inkjet printing; rigorous environmental conditions are required including high temperature (100–200 °C), no oxygen, and no water.^12^ In addition, the toxicity of lead limits its application in the field of anti-counterfeiting.^13–17^ Therefore, the monodisperse spherical lead-free perovskite nanocrystals will play an important role in the field of anti-counterfeiting.

The spray method has been considered as an alternative to the thermal injection method,^18–20^ but it has not been widely used because of its high cost, adverse environmental impact and difficulty in controlling the size distribution of the prepared nanocrystals. In comparison with the hot injection method, the ligand-assisted reprecipitation method at room temperature can produce nanocrystals with good dispersion degrees and regular shapes. In 2016, Li^21^*et al.* first prepared colloidal nanocrystals by ligand-assisted reprecipitation. The morphology of nanocrystals can be controlled in the process of reprecipitation by transforming organic acids and amine ligands.^22^ Additionally, toxic organic acids and amines can be replaced with the non-toxic K30 polyvinylpyrrolidone (PVP).^23^ However, the agglomeration of nanocrystals is still a problem that hinders their application.

Furthermore, the substitution of Pb^2+^ with a monovalent metal cation (Na^+^, K^+^, and Ag^+^) and a trivalent metal cation (Bi^3+^ and In^3+^) is the main strategy, which can maintain the perovskite structure without disrupting the charge balance and also replace the toxic lead element. Recently, lead-free double perovskite nanocrystals have become a hot research topic in the field of luminescent materials.^24–31^

In the present work, spherical Ag^+^ doped Cs_2_KBiCl_6_ nanocrystals were prepared by a generalized ligand-assisted reprecipitation method at room temperature using non-toxic K60 PVP as the ligand. This method is energy-efficient and environmentally friendly. Because of the steric hindrance effect ascribed to the considerable molecular weight (220 000) of K60 PVP, the prepared nanocrystals have good dispersion. The spherical and well-dispersed nanocrystals are easier to inkjet print. The doped silver creates spatially localized electron and hole states in the crystal,^32^ which lowers the transition barrier of Bi^3+^ (^1^S_0_ → ^3^P_1_), and exhibits an orange emission centered at 600 nm under the excitation of 365 nm ultraviolet light. Due to their good dispersion, spherical shape and nanoscale size, the obtained Cs_2_KBiCl_6_ nanocrystals are suitable for inkjet printing anti-counterfeiting patterns. The dispersed spherical Ag^+^ doped Cs_2_KBiCl_6_ nanocrystals were blended with PDMS adhesive and its curing agent to produce a printable ink, which can be used for inkjet printing of anti-counterfeiting patterns.

## 2 Experimental

### 2.1 Raw materials

Cesium chloride (CsCl, 99.99%), potassium chloride (KCl, 99.99%), bismuth acetate (C_6_H_9_BiO_6_, 99.99%), silver acetate (C_2_H_3_O_2_Ag, 99.95%), *N*,*N*-dimethylformamide (C_3_H_7_NO, 99.9%), and isopropanol (C_3_H_8_O, 99.9%) were purchased from Aladdin, K60-type polyvinylpyrrolidone ((C_6_H_9_NO)*n*, average molecular weight 220 000) was purchased from Macklin, and hydrochloric acid (HCl, 36%) was purchased from Chengdu Kelong Chemical. All chemicals were used as is.

### 2.2 Ag^+^ doping Cs_2_KBiCl_6_ synthesis of nanocrystals (using Cs_2_K_0.95_Ag_0.05_BiCl_6_ as an example)

Ag^+^ doped Cs_2_KBiCl_6_ nanocrystals were synthesized by a ligand-assisted reprecipitation method at room temperature. Typically, 2 mmol of CsCl, 0.95 mmol of kCl, 0.05 mmol of C_2_H_3_O_2_Ag, 1 mmol of C_6_H_9_BiO_6_, and 1 g of K60 PVP were dissolved in a solvent mixture including 100 ml of DMF, 20 ml of purified water, and 5 ml of concentrated hydrochloric acid, and then stirred at room temperature for about 1 h. After the reactants had completely dissolved, the above solution was injected into 375 ml of stirring isopropyl alcohol and stirred for about 10 min. The resulting turbid liquid was centrifuged at 8000 rpm for 5 min, the sediment was retained, and then the precipitate was re-dispersed in 5 ml of isopropanol.

### 2.3 Preparation of ink and inkjet printing of anti-counterfeiting patterns

5 ml of Cs_2_K_0.95_Ag_0.05_BiCl_6_ nanocrystal dispersion (concentration: 0.4 mmol ml^−1^) was mixed with 5 ml of PDMS (Brand D, model: DC184) glue and 0.5 ml of curing agent. Then the mixture is thoroughly stirred to ensure uniform dispersion and placed in an oven and heat at 100 °C for 3 hours, which is crucial to achieve the desired viscosity and printability of the ink. Then the prepared printable ink was transferred into the printing needle tubing of a high-viscosity paste direct writing micro-electronic printer (Brand V) and loaded without any air bubbles or contamination. Then, the desired pattern was selected to be printed and the printing parameters were carefully set on the printer's control panel, which is vital for achieving accurate and precise printing. Then, the inkjet printing process was carried out, depositing the ink onto the base material (glass) according to the selected pattern and printing parameters. Then, the printed glass substrate was placed in an oven and baked at 100 °C for 15 hours.

### 2.4 Characterization

The XRD patterns of the samples were collected on a TD-3500 X-ray diffractometer (SmartLab, Rigaku, Japan) using a Cu K_α_ radiation source (*λ* = 1.5406) with an operating voltage of 30 kV and a tube current of 20 mA. The elemental composition of the sample was measured using inductively coupled plasma mass spectrometry (Agilent 5110, USA). TEM images were taken on a transmission electron microscope (JEM-2100F, Jeol, Japan). The absorption spectra of the samples in the range of 200–600 nm were recorded using a UV-VIS-NIR spectrophotometer (UH5700, Hitachi, Japan). The photoluminescence (PL) and excitation spectra were recorded on a spectrophotometer (F-7000, Hitachi, Japan). PL decay lifetime and fluorescence quantum yield (PLQY) were determined on a fluorescence spectrometer (FLS980, Edinburgh, UK).

## 3 Results and discussion

**Fig. 1:**
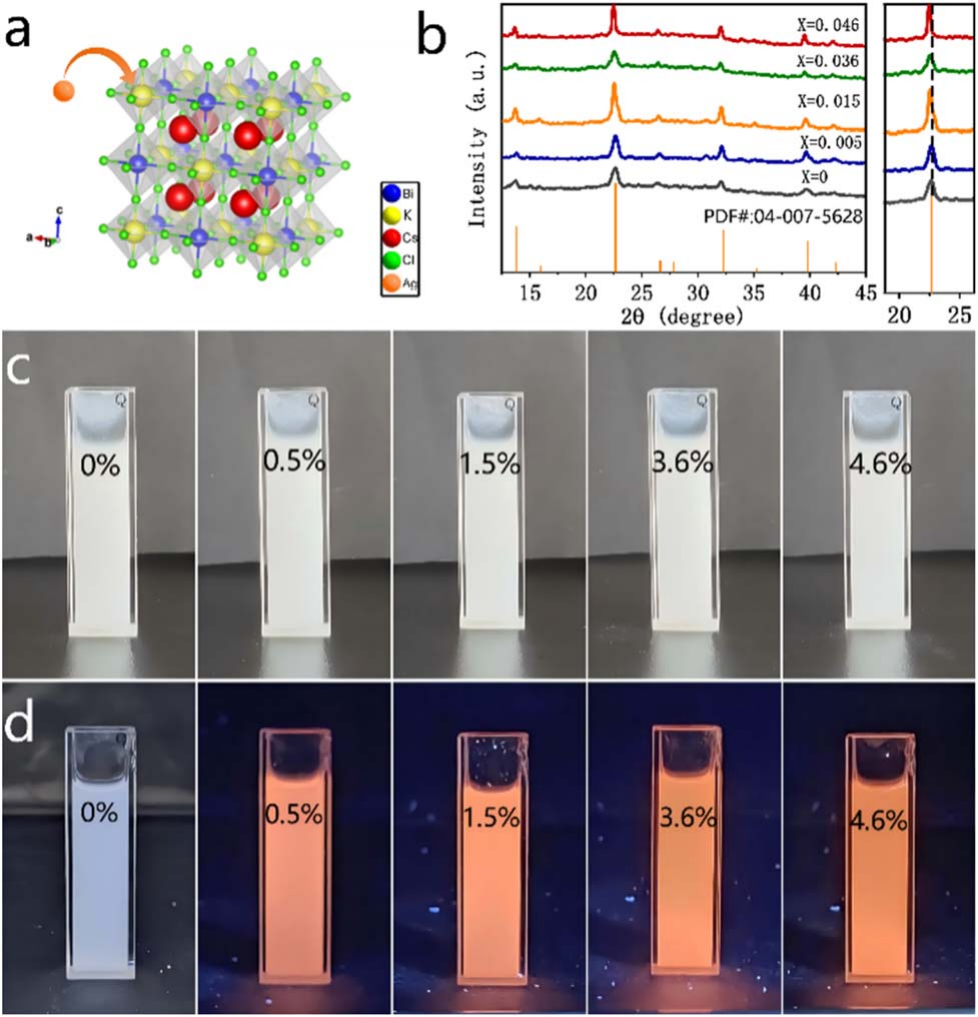
(a) Crystal structure of silver ion doped Cs_2_KBiCl_6_. (b) XRD patterns of Cs_2_K_1−*x*_Ag_*x*_BiCl_6_ (*x* = 0, 0.005, 0.015, 0.036, 0.046) nanocrystals. (c) Photos of Cs_2_K_1−*x*_Ag_*x*_BiCl6 (*x* = 0, 0.005, 0.015, 0.036, 0.046) nanocrystal dispersion liquid under natural light. (d) Photos of Cs_2_K_1−*x*_Ag_*x*_BiCl_6_ (*x* = 0, 0.005, 0.015, 0.036, 0.046) nanocrystal dispersion liquid under ultraviolet light.

A model of the silver doped Cs_2_KBiCl_6_ double-perovskite structure is shown in Fig. 1a. In this structure, [KCl_6_]^3−^ and [BiCl_6_]^3−^ octahedra form a three-dimensional structure, with Cs atoms occupying the interstices of the octahedra, which contributes to structural stability. Silver ions are doped to substitute potassium ions. The X-ray diffraction patterns of the Cs_2_KBiCl_6_ and Ag^+^-doped Cs_2_KBiCl_6_ nanocrystals are presented in Fig. 1b. As the doping level of silver ions increases, the XRD diffraction peak exhibits a maximum leftward shift of 0.18°, indicating a slight expansion of the lattice spacing due to doping. This may be attributed to the fact that the lattice spacing expansion caused by vacancy defects and structural distortions induced by doping counteracts the lattice spacing contraction resulting from the substitution of potassium ions by silver ions. The following table presents the actual doping amounts of silver element in the samples, as determined by ICP analysis (Table 1).

**Table 1:** The mass fraction and actual doping amount of silver element in the sample

Sample no.	Silver ion mass fraction	Silver ion substance fraction
1	0.00%	0.0%
2	0.07%	0.5%
3	0.22%	1.5%
4	0.53%	3.6%
5	0.68%	4.6%

The prepared Cs_2_KBiCl_6_ and Ag^+^-doped Cs_2_KBiCl_6_ nanocrystals appear white under natural light (Fig. 1c). The Cs_2_KBiCl_6_ nanocrystals do not exhibit any considerable luminescence under 365 nm ultraviolet (UV) excitation, whereas the Ag^+^-doped Cs_2_KBiCl_6_ nanocrystals show the orange emission under the same excitation conditions (Fig. 1d).

**Fig. 2:**
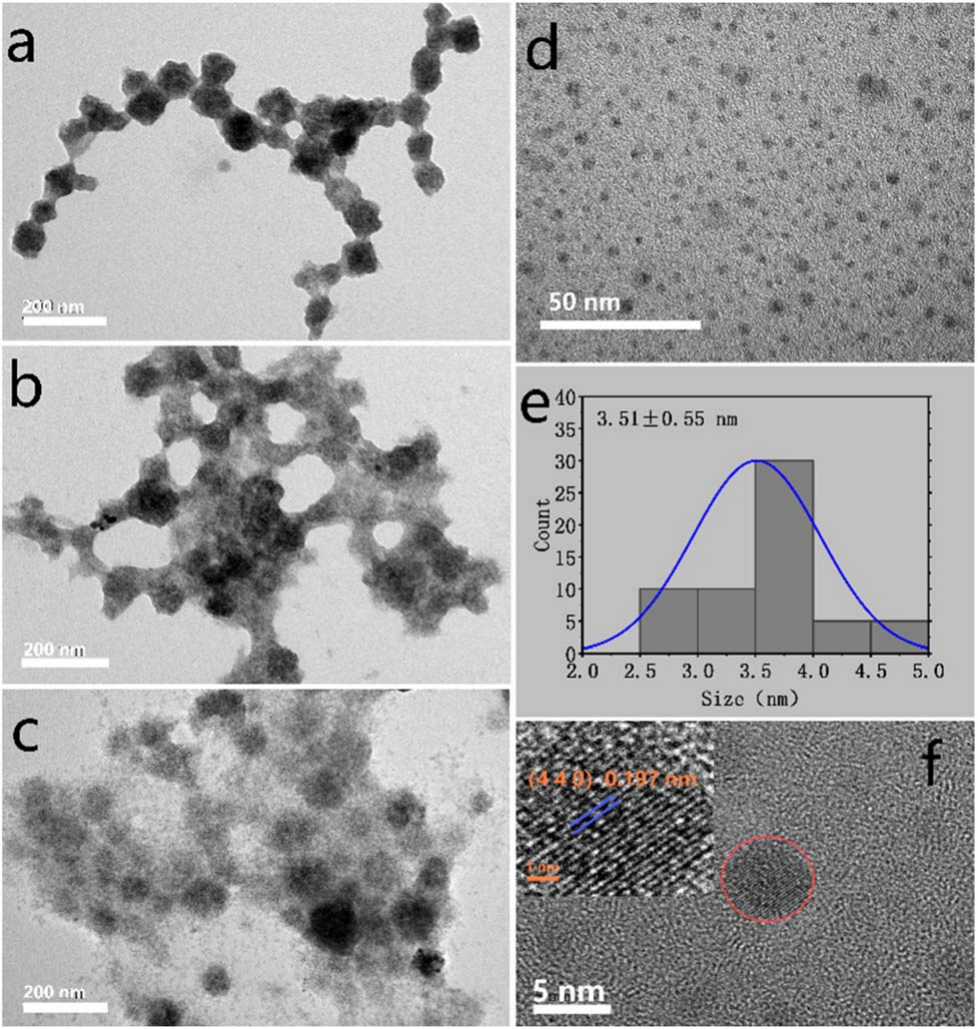
(a–c) TEM images of nanocrystals prepared using different ligands with molecular weights of 8000, 58 000 and 220 000, respectively. (d) Amplified TEM images of Cs_2_K_0.954_Ag_0.046_BiCl_6_ nanocrystals. (e) Size distribution of Cs_2_K_0.954_Ag_0.046_BiCl_6_ nanocrystals. (f) High-resolution TEM image of a Cs_2_K_0.954_Ag_0.046_BiCl_6_ nanocrystal.

Herein, K16 PVP (molecular weight: 8000), K30 PVP (molecular weight: 58 000) and K60 PVP (molecular weight: 220 000) were used as ligands. The Cs_2_KBiCl_6_ nanocrystals were synthesised under the same concentration (1 g mmol^−1^ matrix). Further, the dispersion degree of nanocrystals was compared, as shown in Fig. 2a–c. Increasing the molecular weight of PVP improved the dispersion of nanocrystals, because PVP prevents nanoparticle aggregation through repulsion induced by its hydrophobic carbon chains that extend into solvents and interact with them, which belong to a steric hindrance effect. Therefore, an increase in the molecular weight of PVP likely intensifies this steric hindrance effect, improving the nanoparticle dispersion degree.

Fig. 2d shows the transmission electron microscopy (TEM) images of Cs_2_K_0.954_Ag_0.046_BiCl_6_ nanocrystals. These nanocrystals exhibit an excellent dispersion degree and a spherical morphology. Their mean diameter is 3.51 nm, with a standard deviation of 0.55 nm (Fig. 2e). The high-resolution TEM analysis of the Cs_2_K_0.954_Ag_0.046_BiCl_6_ nanocrystals reveals highly crystalline lattice fringes and a lattice spacing of 0.197 nm, which is slightly larger than the lattice spacing of the (4 4 0) crystal faces of Cs_2_KBiCl_6_ nanocrystals (0.196 nm) (Fig. 2f).

**Fig. 3:**
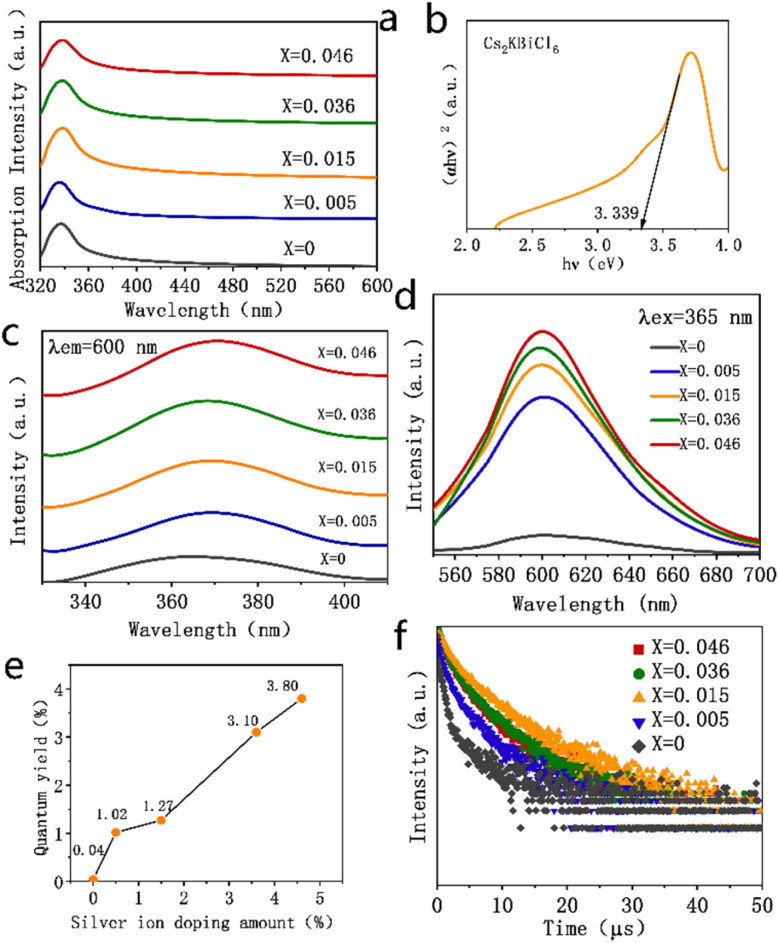
(a) UV-Vis absorption spectra of Cs_2_K_1−*x*_Ag_*x*_BiCl_6_ (*x* = 0, 0.005, 0.015, 0.036, 0.046) nanocrystals. (b) Tauc diagram of Cs_2_KBiCl_6_ nanocrystals. (c) PLE spectra of Cs_2_K_1−*x*_Ag_*x*_BiCl_6_ (*x* = 0, 0.005, 0.015, 0.036, 0.046) nanocrystals. (d) PL spectra of Cs_2_K_1−*x*_Ag_*x*_BiCl_6_ (*x* = 0, 0.005, 0.015, 0.036, 0.046) nanocrystals. (e) PLQY values of Cs_2_K_1−*x*_Ag_*x*_BiCl_6_ (*x* = 0, 0.005, 0.015, 0.036, 0.046) nanocrystals. (f) Photoluminescence attenuation scatter plot of Cs_2_K_1−*x*_Ag_*x*_BiCl_6_ (*x* = 0, 0.005, 0.015, 0.036, 0.046) nanocrystals.

Fig. 3a presents the absorption spectra of the Cs_2_KBiCl_6_ and Ag^+^-doped Cs_2_KBiCl_6_ nanocrystals. The absorption peak at 335 nm observed for the Cs_2_KBiCl_6_ nanocrystals is attributed to the 6s^2^ → 6s^1^p^1^ transition of Bi^3+^ within [BiCl_6_]^3−^. The absorption intensity increases gradually with the Ag^+^ doping amount, accompanied by a red shift of the absorption peaking from 335 to 340 nm. In this study, the optical band gap of the Cs_2_KBiCl_6_ nanocrystals was determined using the absorption spectra and Tauc's formula, which is given below.

1 (*α*ℏ*ν*)^*n*^ = *A*(ℏ*ν* − *E*_g_),

where *α* represents the absorption coefficient, ℏ*ν* is the photon energy (ℏ is Planck's constant and *ν* is the photon frequency), *E*_g_ is the optical band gap and *A* and *n* are constants. For direct band gaps, *n* = 2, while for indirect band gaps, *n* = 1/2. Using this formula, the Cs_2_KBiCl_6_ nanocrystals are calculated to have an indirect optical band gap of 3.339 eV (Fig. 3b). The optical band gaps for the Ag^+^-doped Cs_2_KBiCl_6_ nanocrystals with 0.5%, 1.5%, 3.6%, and 4.6% doping are found to be 3.303, 3.260, 3.194 and 3.171 eV (Fig. S1†), respectively. These results indicate that the optical band gap decreases with increasing Ag^+^ doping amount, with a reduction of only 5%. This suggests that Ag^+^ doping does not substantially change the optical band gap of the Cs_2_KBiCl_6_ nanocrystals. Absorption exhibited by the Cs_2_KBiCl_6_ and Ag^+^-doped Cs_2_KBiCl_6_ nanocrystals can be attributed to the 6s^2^ → 6s^1^p^1^ transition of Bi^3+^ within [BiCl_6_]^3−^. In the excited state 6s^1^p^1^, only the ^3^P_1_ state allows for the partial relaxation of transition prohibition due to spin–orbit interactions, enabling an electronic transition from the ground state 6s^2^ (^1^S_0_ → ^3^P_1_).^33^ After silver doping, the localized electron and hole states are produced in the crystal, which further relaxes the transition barrier and increases the absorption strength.^32^

The photoluminescence excitation (PLE) spectra of Cs_2_KBiCl_6_ nanocrystals were monitored at 600 nm, exhibiting a characteristic excitation peaking at 365 nm due to the ^1^S_0_ → ^3^P_1_ transition of Bi^3+^. For the Ag^+^-doped Cs_2_KBiCl_6_ nanocrystals, this excitation peak is red-shifted to 370 nm, as shown in Fig. 3c. Under 365 nm UV excitation (Fig. 3d), the Cs_2_KBiCl_6_ and Ag^+^-doped Cs_2_KBiCl_6_ nanocrystals exhibit orange emission peaking at 600 nm, which arises from the self-trapped exciton emission of [BiCl_6_]^3−^, attributed to the ^3^P_1_ → ^1^S_0_ transition of Bi^3+^. The emission intensity gradually increases with an increase in the Ag^+^ doping amount, aligning with the trend that Ag^+^ doping substantially increases the fluorescence quantum yield of the Cs_2_KBiCl_6_ nanocrystals. The fluorescence quantum yield of undoped Cs_2_KBiCl_6_ nanocrystals is 0.04%. In contrast, for the Cs_2_KBiCl_6_ nanocrystals doped with 0.5%, 1.5%, 3.6%, and 4.6% Ag^+^, the fluorescence quantum yields are 1.02%, 1.27%, 3.10% and 3.80%, respectively, which represents an increase in the fluorescence quantum yield by a factor of up to 94 times with doping, as shown in Fig. 3e. The ^3^P_1_ → ^1^S_0_ transition of Bi^3+^ is forbidden in the Cs_2_KBiCl_6_ nanocrystals, leading to an extremely low carrier concentration upon UV excitation, which results in an extremely low fluorescence quantum yield. After silver doping, spatially localized electron and hole states are generated in the crystal, which relax the transition barrier,^31^ considerably augmenting the carrier concentration under UV excitation and thus substantially increasing the fluorescence quantum yield. As the Ag^+^ doping amount increases, the relaxation of inhibitions is more obvious, increasing the success rate of the transition. This increases the carrier concentration within the nanocrystal under UV excitation, further augmenting the fluorescence quantum yield.

The luminescence decay curves of the Cs_2_KBiCl_6_ and Ag^+^-doped Cs_2_KBiCl_6_ nanocrystals are shown in Fig. 3f. These decay curves were fitted with a double exponential function.

2 *I*(*t*) = *A*_1_ exp(−*t*/*τ*_1_) + *A*_2_ exp(−*t*/*τ*_2_),

where *I*(*t*) is the fluorescence intensity as a function of time *t*, *A*_1_ and *A*_2_ are constants representing the relative contributions of two different decay modes and *τ*_1_ and *τ*_2_ are two distinct fluorescence lifetimes. The average fluorescence lifetime, *τ*_ave_, was calculated using the following equation.

**Fig. 4:**
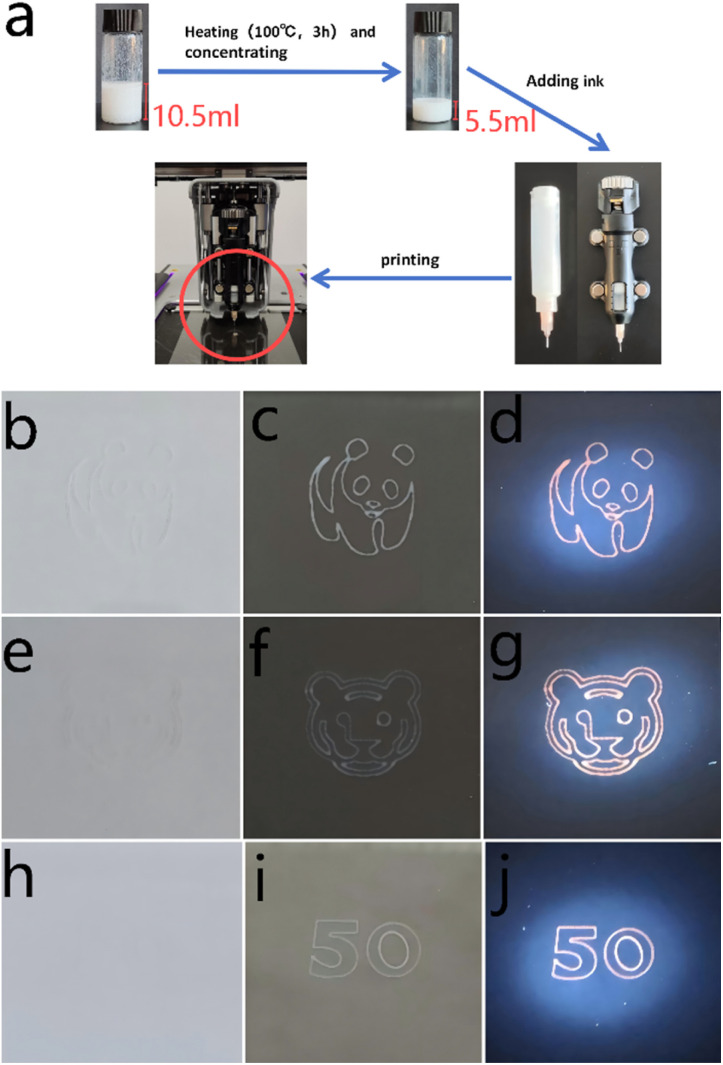
(a) Diagram of the printing process. (b–j) The panda, tiger, and “50” patterns under natural light on a white background, under natural light on a black background, and under ultraviolet light on a black background.

3 *τ*_ave_ = (*A*_1_*τ*_1_^2^ + *A*_2_*τ*_2_^2^)/(*A*_1_*τ*_1_ + *A*_2_*τ*_2_),

where *A*_1_ and *A*_2_ correspond to the relative magnitudes corresponding to the fluorescence lifetimes *τ*_1_ and *τ*_2_, respectively. The luminescence decay curves of the Cs_2_K_1−*x*_Ag_*x*_BiCl_6_ nanocrystals are shown in Fig. 4f. The calculated average fluorescence lifetimes for the Cs_2_K_1−*x*_Ag_*x*_BiCl_6_ nanocrystals are 1.88, 2.42, 4.50, 2.88 and 2.69 μs (Fig. S2†), corresponding to increasing Ag^+^ doping levels. Doping relaxes the transition forbiddenness, thereby increasing the success rate of transitions and consequently extending the fluorescence lifetime. However, an increase in doping concentration leads to a higher overall probability of transition recombination and enhances lattice rigidity, both of which contribute to a reduction in fluorescence lifetime. Due to the combined effects of these factors, the fluorescence lifetime does not exhibit a monotonic change but rather shows a trend of initial increase followed by a decrease.

The nanocrystals synthesised in this investigation exhibit distinct colours when exposed to ultraviolet and visible light, rendering them suitable for applications requiring anti-counterfeiting capabilities. A printable ink imbued with anti-counterfeiting properties was formulated by amalgamating Cs_2_K_0.954_Ag_0.046_BiCl_6_ nanocrystals, which were prepared utilising D brand PDMS adhesive alongside its corresponding curing agent. The schematic representation of this process is depicted in Fig. 4a. Fig. 4b–4j illustrate the printed patterns as observed under varying conditions: the panda, tiger, and “50” patterns under natural light on a white background, under natural light on a black background, and under ultraviolet light on a black background.

## 4 Conclusion

In summary, a generalised ligand-assisted reprecipitation technique was employed at ambient temperature, wherein steric hindrance engineering was utilised to optimise the dispersion characteristics of nanocrystals through the variation of ligand types, resulting in the synthesis of spherical Ag^+^-doped Cs_2_KBiCl_6_ nanocrystals, which maintained an identical double perovskite structure and exhibited excellent dispersibility. The Cs_2_K_0.954_Ag_0.046_BiCl_6_ nanocrystals possessed an average diameter of 3.51 nm. Excited with 365 nm ultraviolet light, the nanocrystals exhibit orange emission peaking at 600 nm, with a fluorescence quantum yield of 3.80%. Subsequently, the nanocrystals were integrated with PDMS adhesive and its corresponding curing agent to formulate a printable ink, which is suitable for inkjet printing of anti-counterfeiting patterns.

## Conflicts of interest

There are no conflicts to declare.

## Supplementary Material

NA-007-D4NA00988F-s001

## Data Availability

Additional experimental data supporting this article are provided in the ESI.† Reasonable requests for additional information can be made to the corresponding author.
