# Supplementary material for: Monodisperse spherical Ag+ doped Cs2KBiCl6 nanocrystals: utilizing steric hindrance engineering for inkjet printing of anti-counterfeiting patterns
Source: Nanoscale Adv. 2025 May 6;7(12):3828–33. doi: 10.1039/d4na00988f (PMC12086864; doi:10.1039/d4na00988f)
Supplement: NA-007-D4NA00988F-s001 [file NA-007-D4NA00988F-s001.pdf]

Supplementary Information (SI) for Nanoscale Advances.
This journal is © The Royal Society of Chemistry 2025

Supplementary material

Monodisperse spherical Ag^{+} doped Cs_2KBiCl_6 nanocrystals: utilizing steric hindrance engineering for inkjet printing of anti-counterfeiting patterns

Shaoli Song,[a] Youlun Zhu, [a] Hanmei Jiang,[a] Huichao He,[a] Qian Yang[a] , Jianbei Qiu[b] and Tao Han*[a]

[a] College of Materials and New Energy, Chongqing University of Science and Technology Chongqing, 401331, China

[b] School of Materials Science and Engineering, Kunming University of Science and Technology Kunming, 650093, Yunnan, P. R. China

*E-mail: danbaiht@126.com

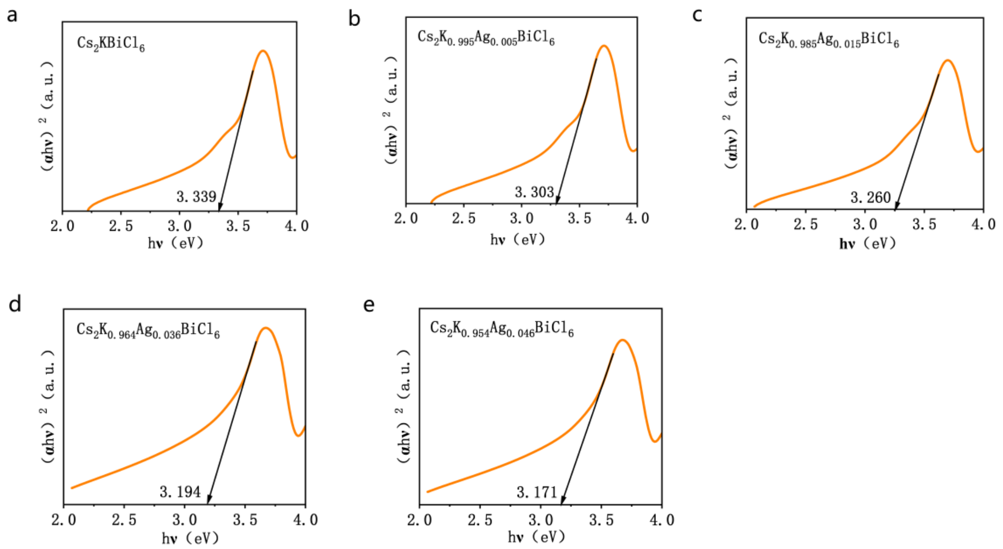

Fig. S1 (a-e) Tauc diagram of $Cs_2K_{1-x}Ag_xBiCl_6$ (x=0, 0.005, 0.015, 0.036, 0.046) nanocrystals.

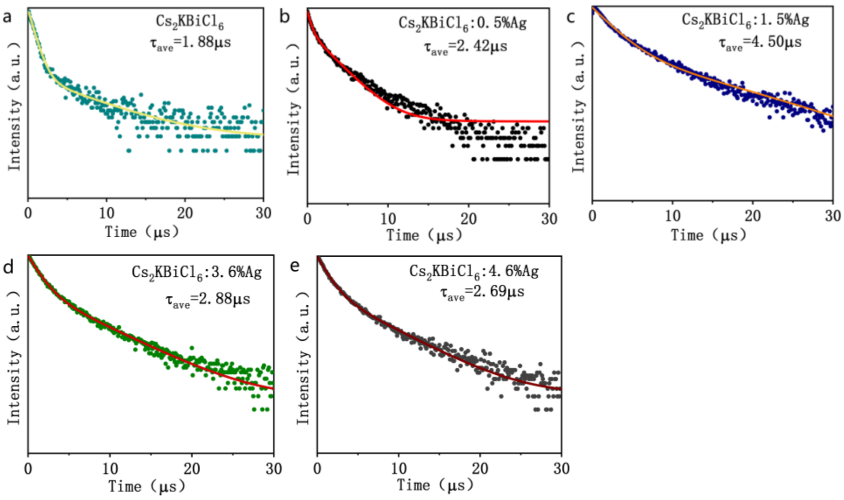

Fig. S2 (a-e) photoluminescence attenuation curves of $Cs_2K_{1-x}Ag_xBiCl_6$ (x=0, 0.005, 0.015, 0.036, 0.046) nanocrystals.
